# Associations between Tactile Sensory Threshold and Postural Performance and Effects of Healthy Aging and Subthreshold Vibrotactile Stimulation on Postural Outcomes in a Simple Dual Task

**DOI:** 10.1155/2016/9797369

**Published:** 2016-04-10

**Authors:** Marius Dettmer, Amir Pourmoghaddam, Beom-Chan Lee, Charles S. Layne

**Affiliations:** ^1^Memorial Bone & Joint Research Foundation, 1140 Business Center Drive, Suite 101, Houston, TX 77043-2740, USA; ^2^Center for Neuromotor and Biomechanics Research, John P. McGovern Campus, 2450 Holcombe Boulevard, Houston, TX 77021-2040, USA

## Abstract

Specific activities that require concurrent processing of postural and cognitive tasks may increase the risk for falls in older adults. We investigated whether peripheral receptor sensitivity was associated with postural performance in a dual-task and whether an intervention in form of subthreshold vibration could affect performance. Ten younger (age: 20–35 years) and ten older adults (70–85 years) performed repeated auditory-verbal 1-back tasks while standing quietly on a force platform. Foot sole vibration was randomly added during several trials. Several postural control and performance measures were assessed and statistically analyzed (significance set to *α*-levels of .05). There were moderate correlations between peripheral sensitivity and several postural performance and control measures (*r* = .45 to .59). Several postural performance measures differed significantly between older and younger adults (*p* < 0.05); addition of vibration did not affect outcome measures. Aging affects healthy older adults' performance in dual-tasks, and peripheral sensitivity may be a contributor to the observed differences. A vibration intervention may only be useful when there are more severe impairments of the sensorimotor system. Hence, future research regarding the efficacy of sensorimotor interventions in the form of vibrotactile stimulation should focus on older adults whose balance is significantly affected.

## 1. Introduction

Aging is known to cause multiple changes in anatomy and physiology of the human body. One significant modification is observed in sensory systems that provide information about body configurations and properties of the external environment. In general, there is a constant decline of sensory functioning and associated sensitivity to stimuli, which begins around the 4th to 5th decade of life with a more rapid decline during the 7th decade. This decline, in addition to loss of cognitive/executive function, leads to problems in sensorimotor processing [1]. Aging of the sensorimotor systems involved in assuring postural stability is a main contributor to the increased prevalence of balance impairments and associated falls in older adults [2–4]. In 2007, there were approximately 1.5 million falls of older citizens (75 years of age and older) reported in the US and approximately 400.000 patients required hospitalization after falls [5]. It is believed that progressive decline of sensory systems function (e.g., plantar mechanoreceptors, vestibular system, muscle spindle afferents, vision) and impairments in proprioceptive-spinal circuits lead to issues regarding the detection of small fluctuations in postural orientation during upright stance [6], which in turn increases the risk for postural balance performance decline and falls [7].

Such deterioration of function often interacts with the affordances posed by specific tasks: Among the major challenges to postural stability is the simultaneous processing of both motor and cognitive tasks. Concurrent postural and cognitive tasking, as often experienced in activities of daily living, may pose a major challenge to older adults, who often exhibit less cognitive capacity (meaning there are fewer available cognitive resources that can be allocated to either of the two tasks) and exhibit generally lower sensorimotor and postural performance. The deterioration in either the cognitive or postural compartment (or both) of such dual-tasks can be interpreted as changes of task processing strategies or prioritization modification.

The modalities of dual-task processing in older adults have been the focus of numerous research endeavors, with major aims being the investigation of executive mechanisms, like concurrent processing modalities, prioritization, and altered behavior patterns due to aging or pathologies [3, 8–14]. Postural control may require cognitive resource itself, and it becomes more difficult at older age and is cognitively more demanding for older people than for younger people [15]. Empirical evidence suggests that aging has a significant effect on processing of attention mechanisms and attention capacity, which is reflected in experimental results showing differences between younger and older participants [6, 14, 16, 17].

Mainly, aging seems to require more attention focused towards the motor control/postural task at hand, whereas complex secondary tasks may lead to increased postural sway compared to younger adults. The specific attention allocation patterns inherent in older adults during sensorimotor/cognitive task processing are based on the complex morphological and functional effects of aging in humans [18, 19]. A common phenomenon related to aging and dual-tasking is the prioritized division of attention and concurrent processing in specific task situations. As an example, older adults more often tend to stop walking when initiating a conversation with a walking companion [3]. Alternatively, often there is a shifting towards more of a motor prioritization that becomes more prominent in demanding postural perturbation tasks and balance threatening situations. Experimental results support the theory of a “posture-first” strategy when facing balance threats, which describes a focus of attention on the postural task in order to prevent falls in older adults, specifically those who are prone to falls [20]. Experiments exposing older adults to postural balance or gait stability threats, like heavy sway, obstacles, or sudden change of surface rigidity, showed a significant prioritization of postural control over cognitive processing either independent of aging or specifically in older adults [21, 22]. Modification of prioritization in specific postural situations can be seen as a plasticity mechanism [3] that is observable in healthy aging and patients suffering from sensory impairments [23].

Results from a number of studies highlight the significance of aging-related degeneration in central and peripheral sensorimotor processing and its effects on postural control [1, 2, 24–28]. Deterioration of the sensory system therefore affects postural control and in turn interacts with attention requirements and modified postural strategies in older adults, specifically in dual-task situations. Younger adults are able to adapt and to compensate for changes of sensory conditions [29]. However, it is also possible to modify and to improve sensory detection and processing, which may assist balance performance in older individuals and patients with neuropathies. A promising approach to achieve augmentation and better function of the somatosensory system is the utilization of interventions based on stochastic resonance (SR). SR is a phenomenon associated with induction of noise into a nonlinear system that is applicable to natural and man-made systems [30]. The term describes the enhancement of neural information transmission and weak stimuli detection when optimal noise is added to the system. The positive impact of SR on system functioning has been observed in a variety of sensory entities and over a wide range of tasks. Hence, tactile receptors and associated touch perception exhibit the beneficial features of SR enhancement. For instance, Well et al. showed that random vibration enhances the detection of weak touch, which has been observed for the foot soles as well [31]. If detection and processing of information can be enhanced via SR, this has significant implications for control of human motion and specifically for human postural control; hence, several studies have investigated the effects of tactile vibration to elicit SR effects on postural and gait performance [7, 32–37]. Considering the potential to enhance peripheral sensory detection and information transmission, it may be possible that a SR-based intervention may have effects on performance dual-task performance. This augmentation of feedback emerging from the foot soles could improve postural control efficiency, which would be associated with less cognitive demand, as potentially expressed either in better cognitive or postural performance in concurrent tasks. An improvement of postural performance in a dual-task has been observed in younger adults [35] but has not yet been investigated for older adults. Such investigations are important since older adults may benefit the most from a potentially balance-enhancing intervention; additionally, the effects of SR may be greater for older adults and those with lower baseline performance [38] or patients suffering from neuropathies [34].

It is still unclear to what extent this improvement of sensory afferent functioning might assist older individuals in performing postural tasks when additional cognitive processing load is added. To our knowledge, no study yet has methodologically investigated whether augmentation of somatosensory feedback does improve dual-task performance in older individuals or whether this intervention could lead to modifications of postural strategies in dual-task conditions.

We designed a study to investigate associations between postural performance and peripheral sensitivity and to investigate effects of aging and SR on healthy adults' postural control and performance. We hypothesized that there would be correlations between sensitivity and postural outcomes and that older adults and younger adults' postural outcomes would differ significantly. We further hypothesized that an SR intervention would affect participants' postural characteristics.

## 2. Methods

This study was conducted according to University of Houston policies concerning the protection of participants in human research. The protocol was approved by the University of Houston Committee for the Protection of Human Subjects (CPHS). All participants in the study provided informed written consent before participation.

### 2.1. Participants

Two groups of participants were recruited for this study, one healthy younger control group and one older experimental group (see Table 1).

Participants were included in the study if they were between the ages of either 20–35 years or 70–85 years. Physical health and cognitive function were initially evaluated based on a modified version of the Physical Activity Readiness Questionnaire and Mini Mental State Exam [39]. Participants were only included if they scored a minimum of 27 on the MMSE and did not report any significant impairments that could put them at risk during the experiment or may affect results. Only those with a BMI below 30 were included in the study. Additionally, participants were only included if they did (at the time of study participation) not use any medication that could interfere with their balance performance. An initial sensory detection test was administered to ensure that older adults displayed an increased tactile threshold at the foot sole, according to criteria described before [7]. Only those individuals who exhibited lower tactile sensitivity were included in the study. Foot length was measured as a requirement for the analysis of limits of stability (time-to-boundary). Demographics of the recruited participant groups are summarized in Table 1.

### 2.2. Equipment

**Figure 1:**
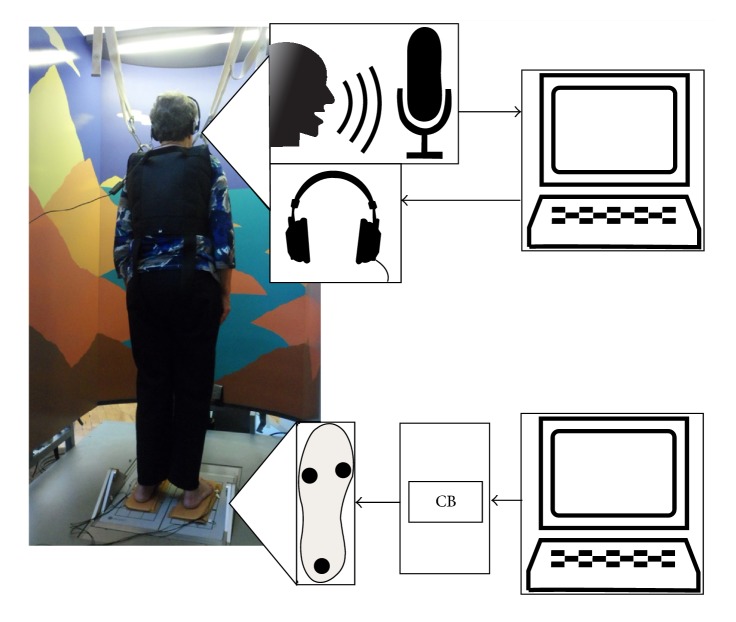
Schematic overview of the experimental setup. Participants received verbal cues to memorize and to recall (1-back task). Custom-made software was used to provide cues and to analyze responses. Vibration was provided via tactor devices embedded in a silicone rubber sole. The soles were connected to a control box (CB) containing the power supply. The control box received commands from custom-made software on a connected computer. Not pictured: A trigger signal from the NeuroCom system was used to initiate stimulus presentation and data collection for the cognitive task.

A custom-made silicone insole was built (Hardness shore 50a) to integrate vibrotactile stimulators (C-2; Engineering Acoustics, FL) that have been used in earlier SR studies [7, 34, 38, 40]. The stimulators were integrated in the insole under the heel, the 1st and 5th metatarsal-phalangeal joint region. The stimulators are magnet motor devices (diameter 30.5 mm, height 7.9 mm, and maximum displacement amplitude of about 0.635 mm) that are connected to a control box including amplifiers and the power supply. In our study, the control box was connected to a PC via a USB cable. A computer-generated white-noise vibration signal band-limited from 1 Hz to about 500 Hz was used as the main mechanoreceptor stimulus. Customized software allowed the modulation of vibration amplitude to adapt it to individually required levels (Figure 1).

To pose a dual-task to participants, custom-made software was used to present verbal cues to the participants during each trial of the experiment via a headset. Verbal responses of the participants were recorded via the headset's microphone, whereas the software used speech recognition to compute both response latency and response accuracy (right/wrong answer).

#### 2.2.1. Center-of-Pressure Data Collection

Center-of-pressure data was assessed using a force plate system (NeuroCom EquiTest, NeuroCom Intl., Clackamas, OR). Force plate data was collected at 100 Hz and processed via software on a connected computer (NeuroCom software version 8.0, NeuroCom Intl., Clackamas, OR).

### 2.3. Procedures

An initial test was conducted to determine if older participants were exhibiting different sensitivity levels related to mechanoreceptors of the foot sole. The testing was based on Semmes-Weinstein filament stimulation according to procedures described elsewhere [7].

After initial testing, participants were accustomed to the vibrating soles, which were adjusted to each participant's shoe size (several silicone strips in the mid-foot section could be added or removed to adjust size). After it was confirmed that the sole fitted well and all stimulators were in place, an initial vibration threshold test was performed. A stimulus intensity level of 90% of perception threshold (100%) has been shown to be effective in SR stimulation experiments, so each participant's threshold was evaluated based on a method of levels [41], to gradually achieve an estimate of each individual's sensory threshold (ST).

In the following experimental trials, participants stood on the force plate for six 20 s trials, with 30 s breaks between each trial (and a two-minute break after three trials). They were instructed to stand quietly during each trial. Vibration conditions were randomized, so that there were three trials including vibration and three without. Due to the vibration amplitude set at 90%, participants were not aware of the current vibration condition.

During each 20 s trial, participants were presented with a series of words via headphones (first word was presented at beginning of each trial, each subsequent word was presented in intervals of 4 seconds). The sequence of words was randomized by the software prior to each trial. Participants were asked to remember and then verbally repeat each word that was presented before the current one (1-back task). They were also asked to try to respond quickly and to speak clearly. Words consisted of the International Radiotelephony Spelling Alphabet, whereas only polysyllabic items were included (24 different items). Initially, participants performed three trials (of 20 s each) of the task in standing position without force plate data collection. These training trials were performed to minimize adaptation to the cognitive task within the experimental trials.

### 2.4. Data Reduction

All outcome measures were computed using customized MATLAB (MATLAB 2012b, The MathWorks, Inc., Natick, MA) scripts and NeuroCom 8.0 software (NeuroCom, Clackamas, OR). Cognitive performance (error rate and reaction time) were assessed via customized software. Outcome measures were averaged for each subject over one block of trials (vibration on or vibration off).

#### 2.4.1. Integrated Time-to-Boundary

Time-to-boundary (TTB) values were generated based on force plate data (measured at 100 Hz). Velocity of the COP in anterior-posterior direction was first calculated based on earlier work [42]. Stability boundaries in the anterior-posterior direction were estimated based on the anterior-posterior limits of a rectangle involving the foot support base and initial foot length measurements. TTB was computed using the formula

(1) $$\begin{array}{c} TTB=\frac{d}{v}, \end{array}$$

whereas *d* is the distance to boundary (*d*), estimated as the distance between the instantaneous COM location and the defined stability limits (boundary) in either given anterior-posterior direction at any moment and *v* is velocity. An integrated area of TTB (iTTB) below a 10 s threshold was then computed for each trial to estimate general stability [42].

#### 2.4.2. Root Mean Square (RMSAP) of COP

RMSAP as a measure of the magnitude of varying quantity was calculated from COP in anterior-posterior plane over the course of each trial of 20 seconds.

#### 2.4.3. Approximate Entropy (ApEn)

ApEn is a nonlinear measure that provides information about the regularity of a time series and has been applied to COP data in a number of different postural studies [43–46]. ApEn calculation was based on computations found elsewhere [47]. ApEn measures were generated using a customized MATLAB code and anterior-posterior COP displacement data for each trial. The data was processed with the following settings in the MATLAB analysis: A series length of 2 (*m* = 2 data points), an error tolerance window of 0.2 times the standard deviation of the respective time series (*r* = 0.2), and a lag value of 10 [48]. A single ApEn value for each trial was generated, which was then used for further statistical analysis and for surrogate analysis (as a necessary precursor to nonlinear/ApEn analysis).

#### 2.4.4. Equilibrium Score (ES)

ES is a measure of postural stability based on hypothetical limits of stability. The formula to calculate ES is

(2) $$\begin{array}{c} ES=\frac{12.5-\left[\theta_{max}-\theta_{min}\right]}{12.5}*100, \end{array}$$

where *θ* are sway angles and 12.5 is the estimated limit of sway (in degrees) for postural control [49]. The score ranges from 0 (a fall) to 100 (no sway).

#### 2.4.5. Anterior-Posterior and Mediolateral Path Length (APPlength and MLPlength)

The summation of all COP displacements over the course of each individual trial was calculated and is expressed through APPlength and MLPlength.

#### 2.4.6. Anterior-Posterior and Mediolateral Maximal COP Excursion (*COPmax*_*A*_ and* COPmax*_*P*_)

The maximal excursion of the COP within each individual trial in both anterior and posterior direction was assessed as an indicator of instability; additionally, the combined maximal excursion in both directions was assessed.

#### 2.4.7. Strategy Score (SS)

SS evaluates movements around the upper body and hips and the lower body (ankles) that are generated for maintenance of postural stability.

The score is based on the formula

(3) $$\begin{array}{c} 1-\frac{{SH}_{max}-{SH}_{min}}{25}*100, \end{array}$$

where SH_max_ and SH_min_ are the shear forces exerted to the force platform. A score of about 100 indicates a strategy based solely on an ankle strategy, and 0 would represent a strategy solely based on hip movements.

#### 2.4.8. N-Back Cognitive Task: Response Time

For evaluation of cognitive performance in the experiment, participants' responses in each trial were analyzed. Data was collected using custom-made software that provided timed presentation of words. The software used Windows-based speech recognition to record both reaction time and correctness of responses during each trial. Correctness was evaluated by the software and defined by the participant correctly verbalizing the earlier (memorized) word right after the presentation of the currently presented one. Correctness was also evaluated by the investigators, who checked each response and noted incorrect responses in an Excel file during each trial. Responses that were incorrect but were corrected immediately by the participants were omitted from the data analysis. The main outcome of the cognitive portion of the experiment was a response time measure (timed at end of response), with response times averaged over all four responses of each trial.

### 2.5. Data Analysis

Statistical analyses of outcome measures were performed using SPSS v. 20 (IBM Corp., Somers, NY). Data are presented as group mean values ± standard deviations (SD). Pearson product-moment correlation coefficients (Pearson's *r*) were computed to investigate associations between tactile sensitivity and postural measures. Mixed-model ANOVA was used to investigate group differences and effects of SR. There was one between-groups factor (age) and one within-group factor (vibration). Analysis was conducted to investigate main effects (vibration and age) and potential interactions (age by vibration). Prior to computation of ANOVA statistics, data were analyzed to evaluate whether all required assumptions (for mixed ANOVA analysis) were fulfilled. Nonnormal distribution of data (as evaluated using Shapiro-Wilk tests with *α*-levels set at 0.05) warranted the use of alternative, nonparametric statistical analysis. Mann-Whitney *U* tests were used for comparisons of pairs of independent samples in this case. Bonferroni adjustment was used to account for multiple comparisons. Significance of statistical comparisons was set at *α* < .05 level.

## 3. Results

Statistical analysis of the initial vibration threshold test revealed that the required vibration amplitude (to achieve 90% of the individual threshold) was significantly larger for the older group than for the younger group, *t*(9.012), *p* = 0.013.

A Pearson product-moment correlation coefficient was computed to assess the relationship between the tactile sensitivity as measured in the beginning of the experiment and different outcomes assessed during dual-tasking. There were several moderate to strong relationships, such as between sensory threshold (ST) and SS (*r* = −.59, *n* = 20, *p* = 0.006), ST and iTTB (*r* = .45, *n* = 20, *p* = 0.047), ST and COPmax_A_ (*r* = .54, *n* = 20, *p* = 0.015), ST and RMSAP (*r* = .57, *n* = 20, *p* = 0.01), ST and APPlength (*r* = .49, *n* = 20, *p* = 0.042), and ST and MLPlength (*r* = .56, *n* = 20, *p* = 0.008).

**Figure 2:**
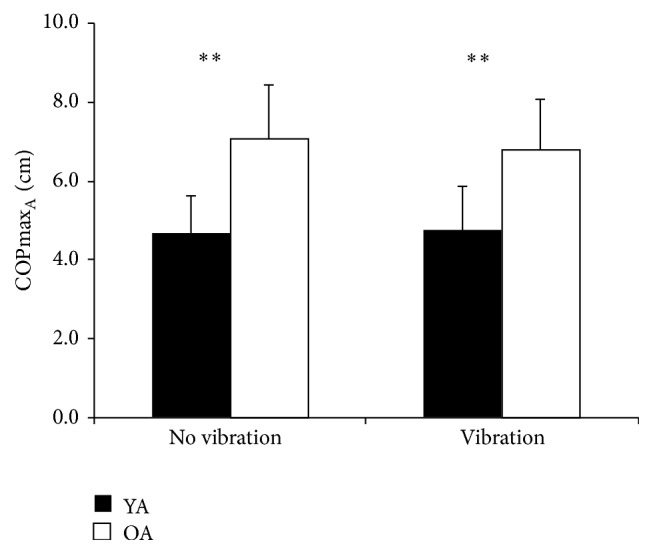
COPmax_A_ means and SD of younger age group (YA) and older age group (OA) without vibration and with vibration. *∗∗* = *p* < 0.01.

**Figure 3:**
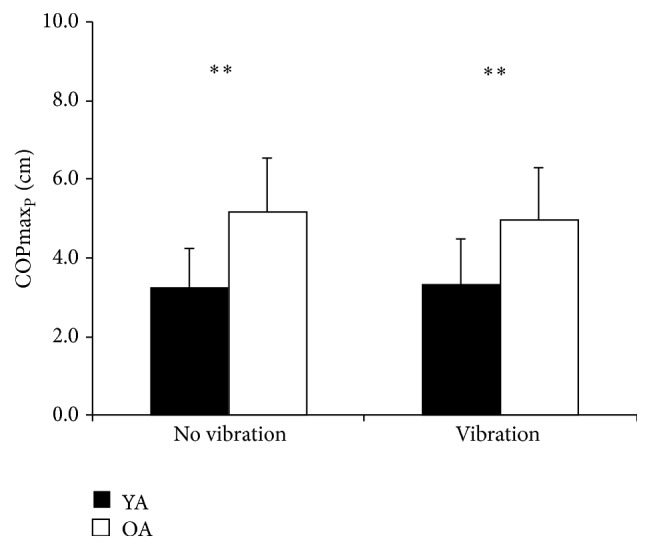
COPmax_P_ means and SD of younger age group (YA) and older age group (OA) without vibration and with vibration. *∗∗* = *p* < 0.01.

**Figure 4:**
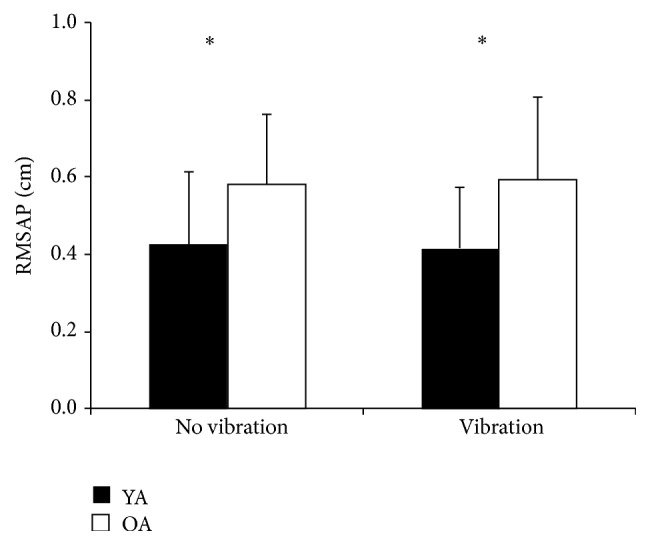
RMSAP means and SD of younger age group (YA) and older age group (OA) without vibration and with vibration. *∗* = *p* < 0.05.

Results from the dual-task experiment are summarized in Table 2. Older and younger participants differed regarding several outcome measures during dual-tasking, that is, COPmax_A_, *F*(1,18) = 17.658, *p* = 0.001 (Figure 2), *η*_*p*_^2^ = .50, COPmax_P_, *F*(1,18) = 12.349, *p* = 0.002, *η*_*p*_^2^ = .41 (Figure 3), RMSAP, *F*(1,18) = 5.956, *p* = 0.025 (Figure 4), *η*_*p*_^2^ = .25, and MLPlength, *F*(1,18) = 5.473, *p* = 0.031, *η*_*p*_^2^ = .233.

**Figure 5:**
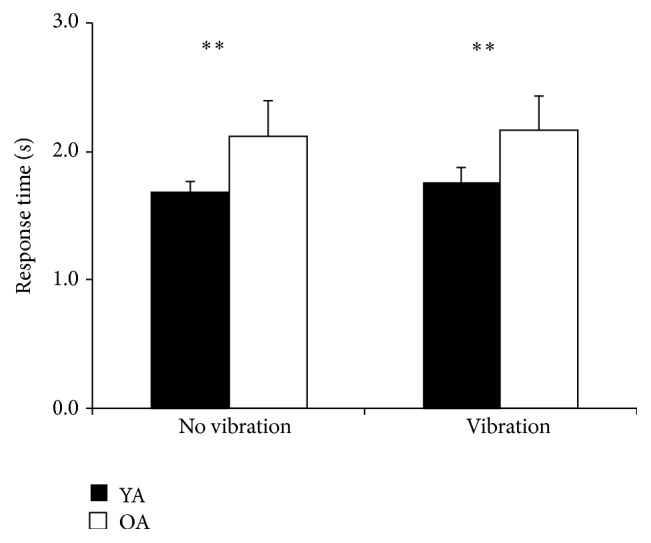
Response time means and SD of younger age group (YA) and older age group (OA) without vibration and with vibration. *∗∗* = *p* < 0.01.

Nonparametric testing showed group differences for APPlength, which differed between older and younger adults both without vibration, *U* = 18, *p* = 0.015, *η*_*p*_^2^ = .29, and with vibration, *U* = 15, *p* = 0.007, *η*_*p*_^2^ = .35. Response time was also significantly different between groups without vibration, *U* = 1.5, *p* < 0.001, *η*_*p*_^2^ = .67, and with vibration, *U* = 6, *p* = 0.001, *η*_*p*_^2^ = .55 (Figure 5). There was no statistical significance for the main factor vibration, and there were no vibration-by-group interactions.

## 4. Discussion

The current experiment was designed to investigate the effects of aging and vibration on dual-task performance and control characteristics. We hypothesized that age and age-dependent loss of peripheral sensory function would be associated with performance and motor control in a dual-task situation and that outcomes would differ between conditions where vibration was either applied or not. We expected that the effects of aging on mental capacity and the sensorimotor decline observed with aging would affect outcomes related to one or both components of the dual-task.

Our hypothesis regarding associations between sensitivity of the foot sole and postural characteristics was confirmed, since several outcomes exhibited moderate to strong correlations with results from initial sensory testing. These findings confirm earlier results regarding the importance of tactile receptor feedback for postural tasks [50–53]. It has been postulated that deterioration of central integrative processes and peripheral sensitivity are contributors to postural control decline [54], and the current experiment provides evidence for this association in a dual-task situation. Additionally, the correlation observed between SS and sensitivity could be interpreted as an expression of sensorimotor adaptation that is required to maintain high levels of balance performance, as those participants who have less sensory feedback are known to adopt a strategy that includes more agonist-antagonist coactivation on the lower leg for higher stiffness with more reliance on hip movements and less ankle movement. Deterioration of sensitivity at the foot soles may be a valuable predictor for balance issues; however, in the current experiment, older adults performed very well regarding the postural task despite increased sensory thresholds. This is remarkable, since older adults exhibiting high function regarding balance tasks or dual-tasking are also affected by sensory decline but potentially adapt to the deterioration by applying different movement strategies, for example, by using different multiple muscle activation patterns [55]. Alternatively, high-functioning older individuals may also have better control through postural reflexes, based on better function of the neuromuscular system in comparison to low-balance performance individuals.

### 4.1. Aging Effects on Balance Control and Performance

Older and younger adults' balance performance differed regarding several but not all postural performance and control measures. This highlights the significance of assessing a number of different COP-based balance parameters, whereas such analyses can assist in the exploration of subtle changes of postural control due to aging, even in individuals whose balance performance is high.

APPlength/MLPlength, maximal COP excursion, and RMS of COP displacement are considered potential indicators of elevated postural instability, so the observed results regarding effects of aging on these outcomes could be interpreted as evidence for less stability in the older group when facing a dual-task challenge. However, considering the otherwise high performance levels of the specific group of older adults in this study, it is possible that path length and COP displacement increases in older adults were based on strategy modification, whereas participants allow for more sway to gather more sensory cues from the lower legs. This exploratory strategy may support the gathering of information [56] and ultimately would improve postural stability.

Results from iTTB and ES analysis did not reveal any group differences. The fact that older adults performed at similar levels to younger adults is unexpected but may be based on the specific task and the group of older adults that served as participants in the study. The *n*-back task performed concurrently in this experiment was designed to divert attention from postural control processes. It has been shown in younger adults that a fairly simple cognitive task (as is the 1-back task applied in our study) can actually lead to improved posture, associated with even less sway than when performing a single-task. The underlying idea is that an internal focus in an overlearned, mostly automatic, and self-organized task like quiet standing (e.g., based on the instruction “stand as quiet as possible”) could interfere with the motor system [57–59]. The cognitive task in our experiment may have shifted the participants' focus towards an external cue, and automatic postural processes ensured maintenance of postural stability. This could explain why our results are contrary to earlier findings that suggested that older adults allowed about 40% increase of instability in favor of maintaining high performance in a concurrent cognitive task [8]. Doumas and colleagues administered a cognitive task that was more demanding than the task applied in the current study. It is likely that increased difficulty of the cognitive task would have shown pronounced age differences, based on increased resource competition. Additionally, the recall task that was applied in the current study differs from some of the tasks used in earlier studies, specifically regarding existing input-output modality pairings. In the current study, a verbal-vocal task was used, which is considered a “compatible” pairing, and which has been shown to be less demanding than dual-tasks including “incompatible” modality pairings [60, 61]. Hence, these tasks require less cognitive effort, which may then be reflected in the postural performance component of an experiment. This highlights the need to consider aspects of modality compatibility in the design of future dual-task studies.

Participants in both groups were able to maintain overall high levels of stability, as evidenced by ES in the range of 90–95, and iTTB values indicating that COP-velocity and overall excursion towards the limits of stability were kept low.

However, we observed a potential trade-off in the cognitive domain. The response times in the *n*-back task administered in this experiment were different between older and younger adults. Older adults required more time to respond in the simple 1-back test compared to younger adults. It is possible that this observation indicates a trade-off between posture and cognitive processing, whereas longer response times were necessary to maintain high levels of postural performance. Alternatively, the trade-off may be a reflection of general aging processes between cognitive features, whereas older adults maintain accuracy of their responses (as observed in our study) while allowing for greater response times. This phenomenon has been observed before whereas, in certain tasks, reaction times are greater in older adults, but response accuracy is the same in comparison to younger adults [62]. This finding was accompanied by higher prefrontal cortex activity in older adults, potentially as a countermeasure to aging processes in the cognitive system [63]. It is possible that this increased cortical activity could make a difference when postural tasks (the primary task) become more difficult, for example, when the visual surrounding or the support surface is sway-referenced. Those tasks would require more conscious control of posture, and aging effects become more pronounced, as has been shown with more demanding secondary tasks [62]. The current results indicate that the older group accepted a trade-off regarding response time in favor of accuracy, a phenomenon that can occur when investigating both measures of accuracy and reaction time [64]. An alternative explanation is that the longer response latency in older adults could stem from prioritization differences due to aging. Participants were given the instruction to respond as quickly as possible, while standing as quiet as possible. Potentially, a higher prioritization was given to the postural task, affecting response times in this group. It is known that older adults have the ability to reallocate cognitive resources according to either instructions or due to strategic decisions, for example, postural stability over cognitive performance [15, 65]. There were no wrong responses in either of the two groups, but it is possible that this would have changed if participants would have focused more on response time than on accuracy. Alternatively, a more demanding 2-back test would have probably caused incorrect responses [62].

In contrast to our initial hypothesis, measures of postural control were not different between younger and older adults, as indicated by similar ApEn and strategy scores. This means that older and younger adults mainly used the same strategic approach to perform the dual-task. Concurrent processing of a cognitive task did not affect older adults in a manner that required them to change the strategy of attention sharing in comparison to younger adults. There arguably was no need for the older group to change the postural strategy compared to younger adults, as would have been evidenced by differences in strategy scores. For more demanding postural/cognitive tasks, more of a top-down strategy, including increased stiffness of the lower-leg musculature [66] and less ankle/more hip rotation, is expected, which is more pronounced in older adults. However, considering the nature of the experimental task in the current study, the need to adopt a top-down approach to postural control was not required.

This idea was supported by results from ApEn analysis. In the current task, temporal dynamics of COP variability were unaffected by age. ApEn seems to be dependent on amount of attention invested in postural control or a secondary task [43, 44]. It can be concluded that the group of older adults included in this study did not adjust postural control to accommodate the requirements posed by the secondary cognitive task, as would have been indicated by changes of our entropy measure. Although ApEn has been shown to detect effects of the addition of a secondary task, even when initial postural sway was minimal [43], it may not be possible to detect age differences when highly functioning older adults are recruited.

### 4.2. Effects of Subthreshold Vibration

We had hypothesized that subthreshold vibration would alter postural performance, control characteristics, or cognitive performance, specifically in the older group. Our initial hypotheses concerning potential effects of subthreshold vibration on dual-tasking, specifically in the elderly, were not confirmed.

Aging requires the allocation of more mental capacity or cognitive resources directed towards postural control or gait [3, 10, 12]. The enhancement of sensory feedback, especially about small excursions of the COP, could have effects on postural stability. This was not the case in the current experiment. The lack of any effects of vibration on either postural or cognitive measures indicates that the subtle enhancement of sensory feedback was not sufficient to affect outcomes. However, the overall high performance levels of the older adults group indicate that there was little necessity for improvement since performance mainly did not differ from the arguably near-optimal performance in the younger group (without vibration). It is unclear if the intervention used in this study could have positive effects if performance levels were lower, for example, in patient populations and recurrent fallers, specifically since SR effects are more pronounced when baseline levels are lower, which has been shown regarding the observed decreases of variability through SR in walking [38]. Additionally, it would be valuable to investigate if the intervention does have an effect in those individuals that suffer from mild or more severe cognitive impairments (and on their cognitive or postural performance).

The connection between postural stability, dual-tasking, and cognitive impairments has been previously identified. As has been concluded from findings in a recent study including a large number of older adults (*n* = 717), it is possible that dual-tasking performance correlates more with fall risk among individuals that suffer from pathological conditions than those who are healthy [66]. The older group recruited for the current study consisted of high-functioning individuals, who live mostly independently and who did not have any cognitive impairment. Earlier research has shown an age-dependent increase in the correlation between cognitive/intellectual abilities and sensorimotor function [19], with an increasingly negative age-dependent correlation between sensorimotor fluctuation and cognitive abilities [67]. The group in our study exhibited higher sensory thresholds but were very similar to the younger group concerning performance and postural control measures. Therefore, the high levels of cognitive function and sensorimotor function retained by this recruited group allowed for performance and control that was similar regarding some performance outcomes, with vibration having no effects on outcomes in either group.

The participant group in this study probably affected results, and considering the exclusion and inclusion criteria that we established, only healthy older adults with high levels of function were recruited. Although those individuals may exhibit higher sensory thresholds and slight decline regarding postural performance compared to younger individuals, differences were relatively small. Further research could aim at investigating the effects of the presented intervention in individuals suffering from mild or more severe cognitive impairment, which could interfere with postural control in dual-tasks. It would be valuable to evaluate whether the intervention can affect performance in those individuals, compared to the current results in healthy older adults.

## 5. Conclusions

Results from the current study indicate specific correlations between tactile sensitivity and postural performance and control in a simple dual-task. The evaluation of tactile sensitivity in older adults for the purpose of prediction of fall risk or postural performance may not be adequate for otherwise high-functioning individuals. Healthy aging affects several postural outcomes in dual-tasking, but the nature of the dual-task and associated modality compatibility may affect results. This has implications for future study designs and the interpretability of results regarding a translation to real-life situations.

A tactile SR intervention may not improve performance if the task is simple, or when participants exhibit high-baseline performance. The application of the technology in a clinical setting may therefore benefit from extensive initial testing, whereas SR-based interventions may be only valuable for certain individuals. Future research should investigate effects of SR in more demanding tasks, in a number of dual-tasks using different sets of modality-mappings, and in individuals suffering from severe sensorimotor impairments.

## Figures and Tables

**Table 1:** Anthropometric characteristics of younger and older participants and sensory threshold expressed as fraction of potential maximum amplitude output of the vibrotactile device.

	*N*	Gender	Height	Weight	Age	Foot length	% of vibration
Younger	10	f 5 m 5	165.6 ± 9.2	148.3 ± 27.2	25.1 ± 2.3	25.4 ± 2.3	2.1 ± 0.6

Older	10	f 8 m 2	165.6 ± 10.6	151.1 ± 35.2	78.6 ± 5.4	25.4 ± 1.6	23.2 ± 21.8

**Table 2:** Means and standard deviations of postural performance, control, and cognitive response time of younger adults (YA) and older adults (OA) with and without vibration.

	ITTB		APPlength (in cm)		MLPlength (in cm)		COPmax_A_ (in cm)		COPmax_P_ (in cm)	
	YA	OA	YA	OA	YA	OA	YA	OA	YA	OA
No vibration	1.4 ± 1.8	2.6 ± 4.2	10.2 ± 2.8	17 ± 7.9	4.1 ± 1.6	6.2 ± 2.1	4.7 ± 1.4	7.1 ± 1.0	3.3 ± 1.2	5.2 ± 1.3
Vibration	1.6 ± 3.1	2.4 ± 3.6	10.3 ± 3.9	16.9 ± 6.8	4.27 ± 2.3	1.6 ± 3.1	4.7 ± 1.3	6.8 ± 1.1	3.4 ± 1.0	5.0 ± 1.1

	RMSAP (in cm)		Strategy score		ES		ApEn		Response time	
	YA	OA	YA	OA	YA	OA	YA	OA	YA	OA

No vibration	.42 ± .2	.58 ± .1	98.5 ± .7	98.0 ± 1.0	92.0 ± 5.8	92.0 ± 2.3	0.62 ± .1	0.68 ± .2	1.7 ± .1	2.1 ± .3
Vibration	.41 ± .2	.59 ± .2	98.5 ± .7	98.0 ± 1.1	93.0 ± 3.0	92.6 ± 1.4	0.62 ± .1	0.69 ± .1	1.8 ± .1	2.2 ± .3
